# Circulating let‐7 Predicts Hepatic Fibrogenesis of 12‐Month Post‐Nucleos(t)ide Analog Treatment in Patients With Hepatitis B Virus

**DOI:** 10.1002/kjm2.70015

**Published:** 2025-03-29

**Authors:** Yi‐Shan Tsai, Po‐Cheng Liang, Yi‐Hung Lin, Tyng‐Yuan Jang, Yu‐Ju Wei, Po‐Han Chen, Jia‐Ning Hsu, Meng‐Hsuan Hsieh, Ming‐Yen Hsieh, Chih‐Wen Wang, Zu‐Yau Lin, Ming‐Lun Yeh, Chung‐Feng Huang, Jee‐Fu Huang, Ming‐Lung Yu, Wan‐Long Chuang, Chia‐Yen Dai

**Affiliations:** ^1^ Hepatobiliary Division, Department of Internal Medicine and Hepatitis Center Kaohsiung Medical University Hospital, Kaohsiung Medical University Kaohsiung Taiwan; ^2^ Health Management Center & Department of Occupational Medicine Kaohsiung Medical University Hospital, Kaohsiung Medical University Kaohsiung Taiwan; ^3^ Faculty of Internal Medicine, College of Medicine and Drug Development and Value Creation Research Center Kaohsiung Medical University Kaohsiung Taiwan; ^4^ School of Medicine, College of Medicine and Center of Excellence for Metabolic Associated Fatty Liver Disease National Sun Yat‐sen University Kaohsiung Taiwan

**Keywords:** hepatic stellate cells, liver fibrosis, microRNAs, nucleos(t)ide analogs, TGF‐β

## Abstract

Chronic hepatitis B virus (HBV) infection is associated with potential complications of liver cirrhosis and hepatocellular carcinoma. To date, there are no effective and noninvasive clinical markers that can predict the risk of liver fibrosis early and accurately in chronic hepatitis B (CHB) patients treated with nucleos(t)ide analogs (NAs). This study aimed to investigate the association of circulating let‐7b/c/g levels with the severity of hepatic fibrosis with a FIB‐4 index of 1.5–2.9 in CHB patients. We conducted a retrospective longitudinal study in patients with CHB after 6 months of NAs therapy to investigate whether serum let‐7b/c/g levels can be monitored as an early biomarker for liver fibrogenesis based on multivariate logistic regression analyses. We also used the hepatic stellate cell line LX‐2 treated with transforming growth factor‐β (TGF‐β) to evaluate the suppression effect of let‐7b/c/g on hepatic fibrogenesis. The study showed that circulating let‐7b/c/g could predict 12 months of antiviral treatment for HBV‐related significant fibrosis (FIB‐4 index ≥ 2.9) at baseline and was significantly negatively correlated with the FIB‐4 score. Moreover, let‐7b/c/g could directly target the TGF‐βR1–3′ untranslated region (3′ UTR) and inhibit TGF‐β induced p‐SMAD2 phosphorylation to reduce α‐smooth muscle actin levels, a fibrogenesis marker in LX‐2 cells. These results confirm that let‐7b/c/g could be a biomarker for monitoring HBV‐induced fibrogenesis.

## 1 Introduction

In Eastern Asia and Africa, 257 million people around the world are estimated to have chronic hepatitis B virus (HBV) infection, and 20 million deaths between 2015 and 2030 will be attributable to acute hepatitis, chronic hepatitis, cirrhosis, and hepatocellular carcinoma (HCC) caused by HBV, with 5 million attributed to HCC alone [1]. HCC accounts for over 80% of primary liver cancer cases and is the fourth most common cause of cancer‐related death globally [2]. Liver cirrhosis (LC) increases the risk of HCC by 59.9‐fold [3]. Therefore, it is necessary to develop new noninvasive testing methods for hepatic fibrosis and medical therapies to halt and reverse the progression of fibrosis and repress the progression to HCC.

During HBV infection, the HBV viral proteins HBX, preS2, and HBsAg enhance transforming growth factor‐β (TGF‐β) expression in the Huh7.5.1 hepatocyte cell line and active LX‐2, which are employed as hepatic stellate cells (HSCs) to induce α‐smooth muscle actin (α‐SMA), TIMP‐1, and Col1A1 expression [4]. Liver‐resident macrophages also produce TGF‐β to mediate activation through the interaction of TGF‐β with its receptor (TGF‐βR) to activate the SMAD2/3/4 pathway [5]. The activation of HSCs is critical for orchestrating the deposition of extracellular matrix (ECM) in normal and fibrotic livers. α‐SMA expression of HSCs is a widely accepted marker that is significantly higher in patients with hepatitis C virus (HCV) or HBV cirrhosis [6].

The areas under the hierarchical summary receiver‐operating characteristic curve (HSROC) for predicting significant fibrosis, severe fibrosis, and cirrhosis using the FIB‐4 index with different cut‐off values are 0.78, 0.79, and 0.89, respectively, in HBV‐infected patients [7]. Liver biopsies of patients with HBV‐related LC should be collected, as abnormal abdominal ultrasound or computed tomography images, which are more invasive, are usually conducted at a late stage [8]. There is a need to develop noninvasive, circulating, and stable biomarkers, or a combination of other factors, to detect LC in patients with early‐stage HBV infection and mild fibrosis (FIB‐4 index 1.5).

MicroRNAs (miRNAs) are non‐coding RNAs that post‐transcriptionally modulate gene expression by affecting the stability and translation of complementary mRNAs. The complementary genome sequences of several mentioned miRNAs (such as the let‐7 family, miR‐122, and miR‐125b‐5p) target mRNA transcripts of HBV and HBV‐related HCC [9]. HBV *PreS2* mRNA is targeted by *let‐7g* [10], and *let‐7* is downregulated by the HBV × protein (HBx) by targeting the signal transducer and activator of transcription 3 [11]. These findings strongly suggest a connection between miRNAs, HBV genomes, and pathogenesis, making miRNAs an alternative target for HBV therapy [12]. Moreover, circulating *let‐7* levels are correlated with hepatic fibrosis progression in patients with CHC [13]. The *let‐7a* suppresses liver fibrosis via the TGF‐β/SMAD signaling transduction pathway in mouse models [14]. On the other hand, patients with HBV with cirrhotic regression have *let‐7d* increasing expression at week 240 post‐NAs treatment [15]. Circulating *let‐7b* and miR‐122 can be useful markers for differentiating early HCC from dysplastic nodules in patients with CHB [16]. Notably, *let‐7g* was downregulated during pre‐ETV treatment and upregulated during post‐ETV treatment (> 2 years) in the liver tissue of patients with CHB, which was lower than in normal liver tissue [17].

Our previous study demonstrated that the circulating levels of *let7b/c/g* represent the let‐7 family [18]. Matsuura et al. used longitudinal analyses in 60 patients with paired liver biopsies and showed that *let‐7* levels in plasma markedly declined over time in parallel with chronic hepatitis C (CHC)‐related fibrosis progression [13]. The circulating levels of *let7b/c/g* have not been determined in patients with CHB. Herein, we conducted a retrospective study to investigate whether serum *let‐7b/c/g* levels can be used to monitor the progression of liver fibrosis. We also confirmed that *let‐7b/c/g* plays a negative role in fibrosis by targeting *TGF‐βR1* and inhibiting TGF‐β induced p‐SMAD2 phosphorylation to reduce the α‐SMA level, a fibrogenesis marker in LX‐2 cells. As a result, circulating *let‐7* miRNAs have tremendous potential as early monitors of fibrogenesis.

## 2 Materials and Methods

### 2.1 Study Participants and Clinical Laboratory Data

This hospital‐based retrospective study conducted from 2003 to 2019 was approved by the ethical committee of Kaohsiung Medical University Hospital (KMUHIRB‐E(II)‐20190405). We retrospectively analyzed 103 naïve patients with CHB who received antiviral treatments according to the Asian Pacific Association for the Study of Liver (APASL) recommendation [19]. Long‐term follow‐up of patients with new development of cirrhosis was classified by ultrasound, computed tomography, or magnetic resonance imaging after antiviral treatment with lamivudine (LAM) or entecavir (ETV) after 6 months. All patients with other types of hepatitis, such as HCV infection, autoimmune hepatitis, primary biliary cirrhosis, sclerosing cholangitis, Wilson's disease, α1‐antitrypsin deficiency, and HCC, were excluded. Blood sampling and medical record review were performed at baseline and 12 months after CHB NAs treatment. Serum markers such as aspartate aminotransferase (AST), alanine transaminase (ALT), international normalized ratio (INR), blood urea nitrogen (BUN), creatinine (Cr), platelets (Plts), alkaline phosphatase, total bilirubin, and albumin (Alb) were measured. HBsAg, HBeAg, and anti‐HBe were examined by enzyme immunoassay (EIA; Abbott Laboratories, North Chicago, IL, USA), and quantitative HBV DNA was performed by the Roche Cobas Apliprep/Cobas Taqman HBV Test (Roche Molecular System, Roche, Branchburg, USA). The availability of FibroScan data for fibrosis staging was limited in the study. Therefore, a FIB‐4 index cutoff value of 2.9 was established to define significant fibrosis [7].

### 2.2 Cell Culture

The LX‐2 cells obtained from Merck Millipore (Darmstadt, Germany) were maintained in DMEM supplemented with 5% fetal bovine serum, 100 U/mL penicillin, and 100 g/mL streptomycin and incubated at 37°C in a humidified atmosphere with 5% CO_2_. Lyophilized TGF‐β1 was obtained from R&D Systems. For TGF‐β treatment, transfected cells were treated with TGF‐β (5 ng/mL) for 24 h.

### 2.3 miRNA Transfection

On the day of transfection, the cells (1.8 × 10^5^) were seeded on six‐well plates and transfected with the mirVana let‐7b/c/g mimic/inhibitor and miRNA mimic/inhibitor negative control purchased from Ambion (Life Technologies) using RNAiMAX as the transfection reagent (Life Technologies) according to the manufacturer's instructions. The cells were washed 24 h after transfection, replenished with a new culture medium, and allowed to grow for 48 h.

### 2.4 Construction of *TGF‐βR1* 3′ Untranslated Region (3′ UTR) Reporter Plasmids

*TGF‐βR1* 3′ UTR was targeted by let‐7 from TargetScan (v8.0; targetscan.org) [20]. The synthesized nucleotides of the *TGF‐βR1* 3′ UTR segment with restriction enzyme sites (5′SpeI and 3′MluI) were cloned into the pMIR‐REPORT luciferase vector (Thermo Fisher Scientific). For reporter assays, LX‐2 cells were transfected with let‐7b/c/g mimic or inhibitor and reporter plasmids (500 ng) carrying wild‐type or mutant sequence and using RNAiMAX (Invitrogen). pEGFP plasmids (200 ng) were cotransfected and used as an internal control. Luciferase activity was measured using the ONE‐Glo luciferase assay kit (Promega).

### 2.5 Quantitative Real‐Time Polymerase Chain Reaction (PCR)

MicroRNAs were extracted from 200 μL of serum using Trizol LS (Thermo Scientific, Waltham, MA, USA). miRNA detection was performed by RT‐qPCR using TaqMan MicroRNA Assays and measured using the 7900 Sequence Detection System (Applied Biosystems). According to our previous study, the expression levels of miRNAs in each sample were normalized to the corresponding spike‐in cel‐39 level [18]. A total of 1 μg cDNA was synthesized from total RNA extracted by RNA purification kits (Zymo Research, Irvine, CA, USA) using a High Capacity cDNA Reverse Transcription Kit (Applied Biosystems, CA, USA). PCR was performed in duplicate using the SYBR Green PCR Master Mix (Applied Biosystems) with the following *TGF‐βR1* sense primer: CAAGCAGAGTACACACAGCAT, *TGF‐βR1* antisense primer: TGCTCCACTTTTAACTTGAGCC [21], *GAPDH* sense primer: GTCTTCACCACCATGGAGAA, and *GAPDH* antisense primer: ATGGCATGGACTGTGGTCAT.

### 2.6 Immunoblot Analysis

Immunoblot analysis was performed to assess protein abundance, as previously described [14]. Anti‐α‐SMA antibody was purchased from Sigma‐Aldrich Inc. Anti‐phospho‐SMAD2 (Ser465/Ser467) (E8F3R) and total SMAD2 (D43B4) antibodies were purchased from Cell Signaling Technology Inc. Anti‐GAPDH antibodies were purchased from Millipore Inc.

### 2.7 Statistical Analysis

Categorical variables were assessed using the chi‐square test with Fisher's exact test. Continuous variables were tested using Student's *t*‐test or analysis of variance, followed by multiple‐comparison post hoc tests (Wilcoxon) to compare the mean differences. Nonparametric Spearman's *ρ* was used to determine correlations between quantitative variables. The tests were two‐sided, and statistical significance was set at a *p*‐value < 0.05. Multivariate logistic regression analyses were also performed to evaluate the odds ratio and 95% confidence intervals for these covariates. All procedures were performed using JMP (SAS Institute, Cary, NC, USA). Graphs were generated using GraphPad Prism software (version 7.0; San Diego, CA, USA).

## 3 Results

### 3.1 Baseline Clinical Characteristics of the Patients

The descriptive characteristics of the 103 patients with HBV at baseline are presented in Table 1. There were no significant differences in sex, body mass index, HBeAg (positive proportion), HB Ag titer, HBV DNA > 2000, and creatinine level. Significant differences were found in white blood cell (WBC) count, platelet count, AST, ALT, bilirubin, albumin, hemoglobin, the rate of hypertension, and serum let7b/c/g expression between the two groups.

**TABLE 1:** Descriptive characteristics of patients infected with HBV at baseline (*N* = 103).

	Total (*N* = 103)	FIB‐4 < 1.5 (*N* = 29)	FIB‐4 index ≥ 1.5) (*N* = 74)	*p*
Sex (male), %	78%	76	78	0.78
Age (years)	47.7 ± 11.8	40.4 ± 9.7	50.6 ± 11.5	< 0.0001
BMI	24.1 ± 3.52	24.4 ± 3.0	23.9 ± 3.7	0.57
HBeAg positive (%)	31.3	42.9	27	0.12
HBs Ag titer		4996 (1339–11,776)	1220 (109–48,680	0.38
HBV DNA > 2000 IU/mL (%)	89	83	92	0.19
WBC, ×10^3^/mm^3^	5.4 (4.4–6.4)	6.3 (5.6–7.8)	4.9 (4.0–6.1)	< 0.0001
Platelet, ×10^3^/mm^3^	161 (119–207)	222 (190–244)	142 (103–175)	< 0.0001
AST (IU/L)	115 (57–294)	63 (33–108)	195 (69–354)	0.0009
ALT (IU/L)	221 (86–491)	126 (49–279)	242 (100–576)	0.0066
Bilirubin (total)	1.2 (0.8–2.1)	0.8 (0.7–1.2)	1.3 (1.0–2.4)	0.0084
Albumin	4.1 (3.6–4.4)	4.4 (4.12–4.5)	3.9 (3.4–4.2)	< 0.0001
Creatinine (mg/dL)	0.9 (0.7–1.0)	0.9 (0.7–1.0)	0.8 (0.7–1.0)	0.99
Hemoglobin (g/dL)	14.1 (12.8–15.0)	14.6 (13.5–15.8)	13.8 (12.7–14.9)	0.038
NAs (ETV/LAM), %	55/48	55/45	53/47	0.82
NAs for 12M_yes%	71	83	64	0.05

*Note*: Continuous variables are expressed as medians (IQR, interquartile range) or the mean ± standard deviation (SD) and used by the Mann–Whitney *U* test; categorical variables are expressed, and chi‐square and Fisher's exact tests were used.

Abbreviations: ALT, alanine aminotransferase; AST, aspartate aminotransferase; BMI, body mass index; SD, standard deviation.

### 3.2 Circulating *let‐7b/c/g* Profiling at Baseline and 12‐Month Antiviral Treatment

**FIGURE 1:**
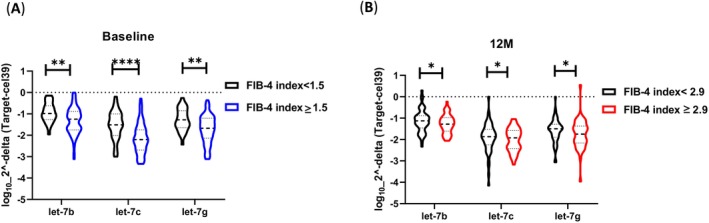
Serum levels of *let‐7b/c/g* in patients with HBV. Relative expressions were normalized to cel‐39 and transformed by log10 at baseline (A) and 12 months after NAs treatment (B). Differences were measured using a Mann–Whitney *U* test, with differences presented as significant with *p < 0.05; **p < 0.01; ***p < 0.001.

Serum *let‐7b* (*p* = 0.003), *let7c* (< 0.0001), and *let‐7g* (*p* = 0.002) levels were significantly lower in the FIB‐4 index ≥ 1.5 compared with the FIB‐4 index < 1.5 at baseline (Figure 1A). After the 12‐month antiviral treatment, *let‐7b* (*p* = 0.02) was also statistically significantly lower in the FIB‐4 index ≥ 2.9 compared with the FIB‐4 index < 2.9 (Figure 1B).

### 3.3 Circulating let‐7b/c/g Expression Levels Correlate With HBsAg, PLT, and FIB‐4 Scores at Baseline After 12 Months

**FIGURE 2:**
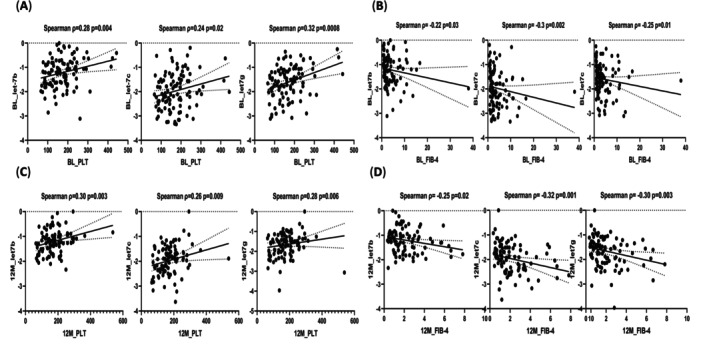
Relationship between differentially expressed circulating *let‐7b/c/g*, PLT, and FIB‐4 levels in patients with HBV infection at baseline and after 12 months. The circulating *let‐7b*, *let‐7c*, and *let‐7g* were positively correlated with PLT and negatively correlated with FIB‐4 at baseline (A and B) and 12 months after NAs treatment (C and D). The dotted line represents the 95% confidence intervals.

At baseline, the correlations between PLT and *let‐7b* (*ρ* = 0.28; *p* = 0.0041), *let‐7c* (*ρ* = 0.24; *p* = 0.0165), and *let‐7g* (*ρ* = 0.32; *p* = 0.0008) are shown in Figure 2A. In Figure 2B, the baseline FIB‐4 score had a significant negative correlation with *let‐7b* (*ρ* = −0.22; *p* = 0.03), *let‐7c* (*ρ* = −0.30; *p* = 0.002), and *let7g* (*ρ* = −0.25; *p* = 0.01). Positive correlations between PLT and *let‐7b* (*ρ* = 0.30; *p* = 0.003), *let‐7c* (*ρ* = 0.26; *p* = 0.009), and *let‐7g* (*ρ* = 0.28; *p* = 0.006) (Figure 2C) as FIB‐4 score negatively correlated with *let‐7b* (*ρ* = −0.25; *p* = 0.02), l*et‐7c* (*ρ* = −0.32; *p* = 0.001), and *let7g* (*ρ* = −0.30; *p* = 0.003) were also observed 12 months after NAs treatment (Figure 2D). The change in *let7b/c/g* and FIB‐4 score from baseline to after 12 months (delta parameters) was not different between patients with ETV and those with LAM treatment (Figure S1).

### 3.4 Circulating *let‐7b/c/g* to Predict 12‐Month FIB‐4 Index ≥ 2.9 (Significant Fibrosis) by Multivariate Logistic Regression Model of Baseline Clinical Parameters

In the LAM group, patients through complete 12 M NAs treatment revealed that (OR: 0.1, 95% CI 0.97–0.99; *p* = 0.003) negatively correlated with a 12‐month FIB‐4 index ≥ 2.9. In the ETV group, baseline WBC (OR: 0.54, 95% CI 0.33–0.89; *p* = 0.009), *let‐7c* (OR: 0.31, 95% CI 0.10–0.91; *p* = 0.03), and *let‐7g* (OR: 0.21, 95% CI 0.06–0.73; *p* = 0.01) were significant predictors of HBV infection in patients with a 12 M FIB‐4 index ≥ 2.9. Multivariate logistic regression revealed that baseline *let‐7g* (OR: 0.23, 95% CI 0.06–0.80; *p* = 0.04) was an independent factor associated with a 12‐month FIB‐4 index ≥ 2.9 in the ETV group (Table 2). We identified fibrosis progression in 13 patients (12.6%) with a Δ FIB‐4 > 0.3 (calculated as the M12‐FIB‐4 index minus the baseline FIB‐4 index). In contrast, 90 patients (87.4%) exhibited regression or remained stable with a Δ FIB‐4 ≤ 0.3. Neither baseline serum let‐7b/c/g levels nor their changes from baseline to M12 were significant in predicting progression, possibly due to the short one‐year follow‐up period and small sample size (Table S1).

**TABLE 2:** Prediction of post‐NAs 12‐month FIB‐4 index ≥ 2.9 by univariate and multivariate logistic regression models of baseline parameters.

Term	LAM		ETV					
	Univariate analysis		Univariate analysis		Multivariate analysis (model 1)		Multivariate analysis (model 2)	
	OR (95% CI)	*p*	OR (95% CI)	*p*	OR (95% CI)	*p*	OR (95% CI)	*p*
BL_WBC	0.43 (0.18–1.01)	0.05	0.54 (0.34–0.86)	0.009	0.54 (0.33–0.89)	0.01	0.56 (0.34–0.92)	0.02
BL_Bil	1.20 (0.84–1.70)	0.32	1.0 (0.70–1.40)	0.97				
BL_Alb	0.52 (0.11–2.38)	0.40	0.32 (0.1–1.26)	0.10				
BL_HGB	0.65 (0.39, 1.09)	0.11	0.74 (0.49, 1.13)	0.17				
NAs for 12M_yes	0.10 (0.01, 0.37)	0.003	0.32 (0.1–1.06)	0.06				
log10_2^‐delta_pre7b	0.62 (0.19, 2.03)	0.43	0.31 (0.09, 1.06)	0.06				
log10_2^‐delta_pre7c	1.21 (0.50, 2.97)	0.67	0.31 (0.10, 0.91)	0.03	0.31 (0.10, 1.05)	0.06		
log10_2^‐delta_pre7g	0.72 (0.23, 2.23)	0.57	0.21 (0.06, 0.73)	0.01			0.23 (0.06, 0.80)	0.04

### 3.5 *Let7b/c/g* Target *TGF‐βR1* 3′ UTR

**FIGURE 3:**
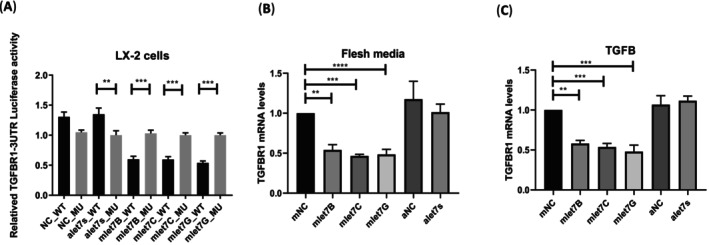
Ectopic expression of *let‐7b* significantly repressed luciferase activity in cells containing wild‐type 3′ UTR regions of TGF‐βR1. Anti‐*let7b/c/g* were reversed to mimic *let‐7* suppression on wild‐type 3′ UTR regions of *TGF‐βR1*, but not mutant‐type 3′ UTR regions of *TGF‐βR1* in human hepatic stellate cell LX‐2 (A). The let‐7b/c/g mimic (50 nM), their inhibitor (50 nM), or NC miRNA (50 nM) were transfected into LX‐2 cells, followed by treatment with fresh media without TGF‐β (B) or with TGF‐β (5 ng/mL) (C) 24 h post‐transfection. RNA was isolated 48 h post‐transfection. The *TGF‐βR1* expression was measured by real‐time PCR. Data are expressed as means ± SEM obtained from three experiments. *p < 0.05; **p < 0.01; ***p < 0.001.

Ectopic expression of *let‐7b* repressed TGF‐βreceptor 1 (*TGF‐βR1*) expression directly by targeting the two let‐7b binding sites in the 3′ UTR in kidney cells [22]. The sequences of wild‐type 3′ UTR and mutated‐type 3′ UTR of *TFGβR1* were constructed into a pMIR‐REPORT vector, and the yellow mark represents the seed region of the *let‐7* binding site (Figure S2). We further identified and confirmed the presence of *let‐7b/c/g* in HSC line LX‐2 cells transfected with 50 nM of mimic *let‐7b/c/g* or anti‐miRNAs and their control scramble. At 48 h post‐transfection, *let‐7b/c/g* directly affected *TGF‐βR1*‐3′ UTR wild‐type but not the mutant‐type reporter (Figure 3A). The *TGF‐βR1* mRNA was significantly downregulated by mimic *let‐7b/c/g* (Figure 3B) or with TGF‐β treatment (Figure 3C).

### 3.6 *Let7b/c/g* Inhibit the Fibrogenesis Marker

**FIGURE 4:**
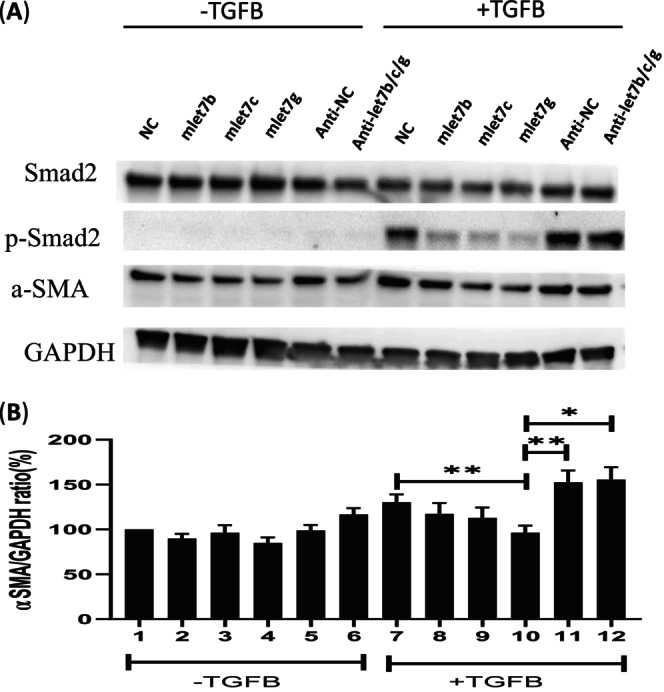
Attenuation of TGF‐β‐induced α‐SMA expression by ectopic let‐7b/c/g expression in LX‐2 cells. α‐SMA and p‐SMAD2 expression were significantly lower in the *let‐7b/c/g* mimic (50 nM final concentration), *let‐7b/c/g* mimic inhibitor (anti‐let‐7s; 50 nM final concentration), or negative control that were transfected into LX‐2 cells followed by treatment with fresh media without TGF‐β or with TGF‐β (5 ng/mL) 24 h post‐transfection. α‐SMA and p‐SMAD2 expression were assessed by immunoblot assay (A). The quantitative α‐SMA expression level was performed using *GAPDH* as an internal control (B). Values are means ± SEM; *n* ≥ 3. *p < 0.05; **p < 0.01; ***p < 0.001.

TGF‐β receptor‐mediated signaling through SMAD2 phosphorylation is widely known [23]. The TGF‐β as a guardian, maintains liver homeostasis [24]. Moreover, serum levels of TGF‐β were significantly increased in patients with CHB compared with healthy controls [25]. The let‐7 family members (*let‐7b/c/d/g/i*) are also downregulated in TGF‐β‐treated mouse mesangial cells (MMCs) [26]. More interestingly, let‐7a is reduced in the liver and serum samples of 12 non‐tumorous patients diagnosed with liver fibrosis and the CCl4‐induced mice fibrosis model [14]. They also found that let‐7a suppresses liver fibrosis via the TGF‐β/SMAD signaling transduction pathway. However, *let‐7b/c/g* levels remain unknown in human hepatic cells. In this study, we tested whether *let‐7b/c/g* protects LX‐2 from Smad2 phosphorylation and fibrogenesis. When LX‐2 was treated with TGF‐β, phosphorylation of SMAD2 (p‐Smad2) and α‐SMA protein levels were significantly increased, as anticipated. After transfection with let‐7b/c/g mimic oligonucleotide, SMAD2 phosphorylation and α‐SMA protein levels were significantly decreased. Conversely, the protein levels of p‐SMAD2 and α‐SMA did not decrease in cells after transfection with the let‐7s inhibitor (anti‐let7b/c/g) (Figure 4).

## 4 Discussion

In this study, we employed patients infected with HBV to demonstrate that *let‐7b/c/g* was associated with the clinical characteristics. Circulating *let‐7b/c/g* expression levels were positively correlated with PLT and negatively correlated with the FIB‐4 index at baseline and 12 months after NAs treatment. The change in *let7b/c/g* and FIB‐4 index from baseline to after 12 months (delta parameters) was not different between patients with HBV treated with ETV and those treated with LAM. Furthermore, *let‐7g* exhibited discriminative odds (OR = 0.23, *p* = 0.04) for the 12‐month FIB‐4 index (≥ 2.9) in patients with HBV, especially with ETV treatment, by multivariate analysis. We also found that *let7‐b/c/g* directly targets TGF‐β receptor1 (*TGF‐βR1*) 3′ UTR and suppresses TGF‐β stimulated SMAD2 phosphorylation (p‐SMAD2) with a decrease of α‐SMA protein level in LX‐2 cells.

Our previous study demonstrated that the circulating levels of *let7 b/c/g* represent the let‐7 family and *let‐7* was positively correlated with serum platelets of patients with CHC [18]. The WBC count was also an independent risk factor associated with a high FIB‐4 index [27], as observed in the present study. High TGF‐β serum levels in patients with chronic HBV infection have been previously observed [25]. Furthermore, immunohistochemical studies by Carpino et al. reported a significantly higher percentage of α‐SMA‐positive HSCs in the HBV cirrhosis group compared with the liver donor group [6]. This process involves complex factors such as TGF‐β and α‐SMA [28]. This is further supported by the recent observation that *let‐7* suppresses CCl4 and bile duct ligation‐induced liver fibrosis by inhibiting hepatocyte apoptosis and TGF‐β production through a *let‐7*/TET3 negative feedback mechanism in mouse hepatocytes [29]. Here, we did not detect serum TGF‐β protein levels owing to degradation in serum long‐period storage. Zhang et al. found that *let‐7a* was reduced in the liver and serum samples of 12 non‐tumorous patients diagnosed with liver fibrosis and a CCl4‐induced mouse fibrosis model via the TGF‐β/SMAD signaling transduction pathway [14]. However, they did not report that *let‐7a* targets the molecular factor that regulates liver fibrosis. We first identified TGF‐βR1 as a potential target of *let‐7b/c/g* in HSCs (LX‐2), and *let7b/c/g* can prevent TGF‐βR1 downstream p‐SMAD2 from reducing α‐SMA expression during in vitro TGF‐β stimulation.

In in vitro HBV‐infected model and HBx‐expressing HepG2 cells, the let‐7 family of miRNAs is significantly downregulated [11]. Moreover, the expression of HBV transcripts, including the preS2 region, is a target of let‐7g [10]. Chen et al. identified four miRNAs, *let‐7c*, *miR‐23b*, *miR‐122*, and *miR‐150*, with significantly increased expression levels in occult hepatitis B infection (OBI) serum compared with healthy control serum [30]. HBV could be transmitted in blood that was positive for HBsAg. Patients with OBI are negative for HBsAg and have normal ALT and AST activities and a low viral DNA loading compared to patients with chronic HBV. Chen et al. identified four miRNAs, *let‐7c*, *miR‐23b*, *miR‐122*, and *miR‐150*, showing a high accuracy level in patients with OBI serum compared to those with control serum [30]. The let‐7 family can inhibit Bcl‐xL expression by inducing apoptosis, which is the cell's natural mechanism for programmed cell death and regulation of tissue homeostasis [31]. We did not include the health group, but we observed that circulating *let‐7b/c/g* expression levels were positively correlated with HBsAg at baseline and after 12 months in patients with HBV (except let‐7b). The let‐7 family might stably reflect the physiological response of liver cells and be regarded as a protective molecule.

When infection is successfully controlled, maturation of T‐cell memory occurs efficiently. HBx has also been implicated in the inhibition of intracellular innate immunity. Patients with CHB have extremely weak HBV‐specific T‐cell responses, which are inversely correlated to viremia levels and express inhibitory molecules like PD‐1, CTLA‐4, and TIM‐3 [32]. PD‐1 is a ligand of PD‐L1, which can be targeted by *let‐7* [33]. Severely exhausted HBV‐specific CD8 T‐cell subsets were detected in a subgroup of patients with CHB [34]. The *let‐7* can regulate the function of adaptive immune cells to affect the differentiation of effector CD8 T cells, which can release effector cytokines and eliminate the infected target cells [35]. Mechanistically, *let‐7* restrains metabolic changes by inhibiting the PI3K/AKT/mTOR signaling pathway to promote memory cells and antagonize terminal differentiation in vivo mouse models [36].

We suspect that a high *let‐7* expression level may modulate the immune response to reduce cytokine TGF‐β. Circulating *let‐7* levels in plasma and extracellular vesicles correlate with hepatic fibrosis progression in chronic hepatitis C [13]. Patients with cirrhotic HBV (baseline Ishak fibrosis score of 5–6) who received 240 weeks of NAs treatment with fibrosis regression had higher *let‐7d* expression [15]. We previously showed that three clusters were obtained from circulating let‐7 family members in patients with CHC and controls. Cluster 1 included let‐*7a/d/e/g*, Cluster 2 comprised *let‐7b* and *let‐7i*, and Cluster 3 comprised *let‐7c/f/miR‐98* [18]. Thus, circulating *let‐7* cluster 1 (*let7a/d/e/g*) may be detected as a stable blood‐based marker for liver fibrosis.

This study had some limitations. First, we did not include a health group. It should be observed whether circulating *let‐7* levels were lower in patients with HBV compared to the healthy group. Second, we did not detect serum TGF‐β protein levels owing to long‐term storage degradation. We limited ourselves to evaluating the relationship between serum TGF‐β and *let‐7* expression. Third, the relationship between *let‐7*, *TGF‐βR1*, p‐SMAD2, αSMA, and PreS2 in the liver tissue of patients with HBV was bound by ethical concerns. In conclusion, circulating *let‐7b/c/g* could be considered independent monitors in patients with HBV as a baseline for predicting liver fibrogenesis.

## Ethics Statement

The study was approved by the Human Research Ethics Committee at Kaohsiung Medical University Hospital (KMUHIRB‐E(II)‐20190405) and followed the ethical guidelines of the Declaration of Helsinki.

## Conflicts of Interest

The authors declare no conflicts of interest.

## Supporting information


**Data S1** Supporting Information.

## Data Availability

The datasets generated and analyzed during the current study are not public but are available from the corresponding author upon reasonable request.
