# Supplementary material for: Circulating let‐7 Predicts Hepatic Fibrogenesis of 12‐Month Post‐Nucleos(t)ide Analog Treatment in Patients With Hepatitis B Virus
Source: Kaohsiung J Med Sci. 2025 Mar 29;41(6):e70015. doi: 10.1002/kjm2.70015 (PMC12199609; doi:10.1002/kjm2.70015)
Supplement: Supplementary file 1 — Data S1 Supporting Information. [file KJM2-41-e70015-s001.docx]

Supplementary file:


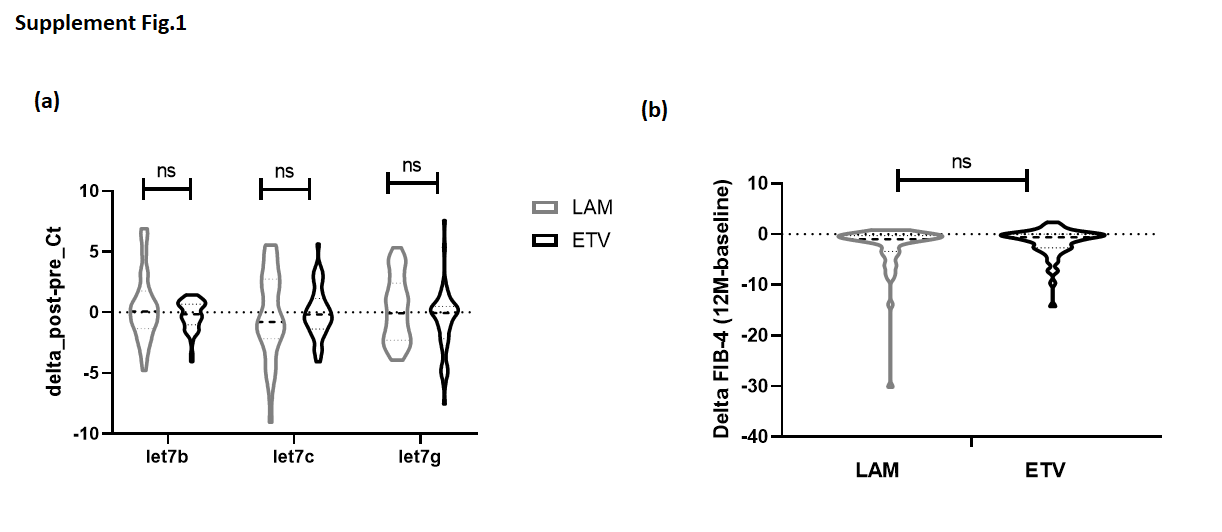


**Supplement Fig.1 Comparing two subgroups divided by NAs treatment (LAM and ETV).** The change in let7b/c/g (a) and FIB-4 score (b) from baseline to after 12 months (delta parameters) was not different between patients with ETV and those with LAM treatment. Differences were measured using a Mann–Whitney U test with differences presented as significant with *p<0.05, and not significant (p>0.05) with “ns”.


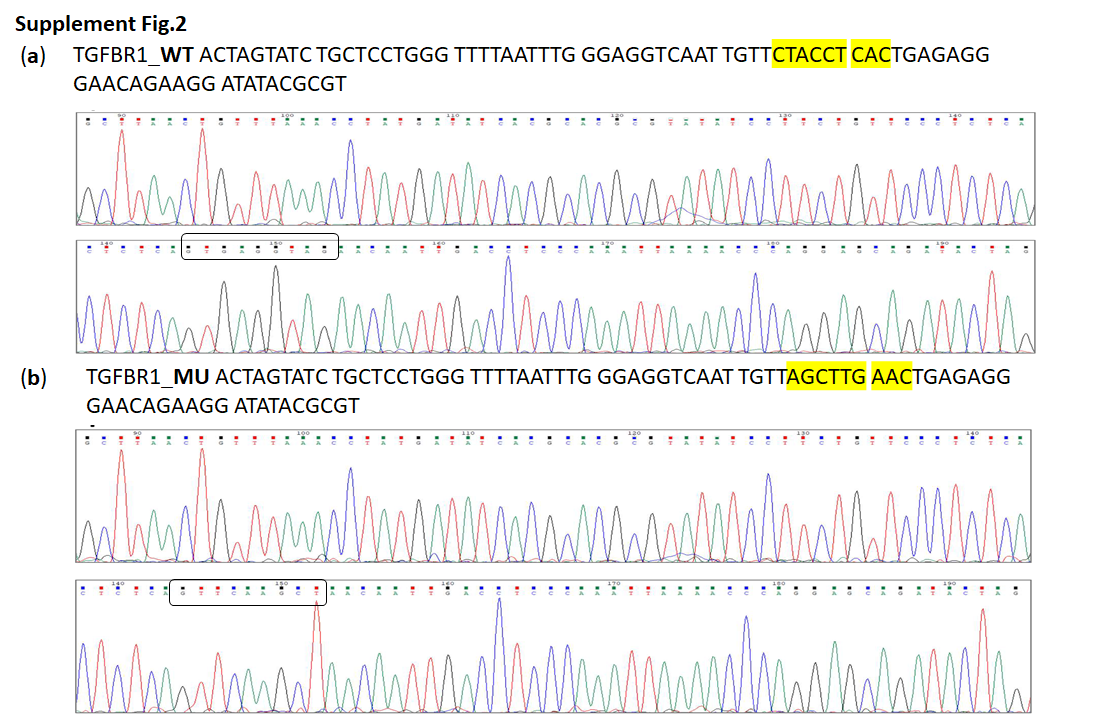


**Supplement Fig.2 *TGF-βR1 3´UTR sequence***

The sequence of wild-type 3’UTR (a) and mutated-type 3’UTR (b) of TFGBR1 were constructed into a pMIR-REPORT vector and the yellow mark represents the seed region of the let-7 binding site. Gene Name: TGFBR1 WT-let7, Gene Size: 79 bp, Vector Backbone: pMIR-REPORT Luciferase, and Cloning Sites: SpeI/ MluI; Gene Name: TGFBR1 MU-let7, Gene Size: 79 bp, Vector Backbone: pMIR-REPORT Luciferase, and Cloning Sites: SpeI/ MluI.

| Table S1. Prediction of Fibrosis Progression (Δ FIB-4 > 0.3) Based on Baseline Serum let-7b/c/g Levels or Their Changes from Baseline to M12 | | | |
| --- | --- | --- | --- |
| Term | Odds Ratio | Lower 95% | Upper 95% |
| pre7b | 0.75 | 0.29 | 1.95 |
| pre7c | 0.63 | 0.28 | 1.42 |
| pre7g | 0.83 | 0.34 | 2.00 |
| post-pre_let7b | 0.99 | 0.37 | 2.67 |
| post-pre_let7c | 0.93 | 0.45 | 1.93 |
| post-pre_let7g | 0.91 | 0.43 | 1.94 |
